# PD155 RedETS Horizon Scanning: Impact In The Decision-Making Process

**DOI:** 10.1017/S0266462324003878

**Published:** 2025-01-07

**Authors:** Janet Puñal-Riobóo, Ignacio López-Loureiro, María del Carmen Maceira Rozas, Beatriz Casal Acción, María José Faraldo Vallés, Margarita Segovia Roldán, Berta Mestre-Lleixà, Gaizka Benguria-Arrate, Tasmania del Pino-Sedeño, Gustavo Mora Navarro, Mar Polo-deSantos, Aurora Llanos Méndez, Jutta Knabe Guerra, Juan Ignacio Martín Sánchez, Maria-Dolors Estrada, Roland Pastells-Peiró, Lorea Galnares-Cordero, Amado Rivero-Santana, Setefilla Luengo-Matos

## Abstract

**Introduction:**

The RedETS horizon scanning (HS) program in Spain is focused on identifying non-pharmaceutical emerging health technologies. HS is organized in three steps: (i) identification using different sources (PubMed, the biomedical press, and others); (ii) screening performed by the HS Working Group and clinicians; and (iii) prioritization using the PriTec tool. This study aimed to evaluate the accuracy of RedETS HS in identifying disruptive emerging technologies for our health system.

**Methods:**

Data from brief files and full reports related to the identified emerging technologies were collected. Full health technology assessment (HTA) reports were also reviewed. The period of analysis was from 2016 to 2023. The information collected included the name, type, category, and indication of the emerging technology and the source of identification. An ad hoc Excel spreadsheet was designed to collect the information. The analysis consisted of a description of the variables and an assessment of concordance between the emerging technologies identified and those with full HTA reports.

**Results:**

There were 338 emerging technologies identified. These technologies were mainly therapeutic (52.1%) or diagnostic (25.7%). In addition, about 45 percent were medical devices and 15.7 percent were in vitro diagnostic tests; imaging comprised 7.4 percent. Most of the emerging technologies were identified through the biomedical press (22.2%), PubMed (23.6%) and industry (20.3%). In a preliminary analysis of these main sources, 31 percent of the technologies identified by HS had full HTA reports, with all of these being identified three years before the HTA.

**Conclusions:**

HS systems might help identify the most relevant technologies for healthcare systems, enabling them to be more ready to incorporate the new technologies. Therefore, HS must be able to detect emerging technologies that will have an impact on the health system. Periodic evaluation of the accuracy of HS programs will improve their impact in the HTA process.

